# Comparison of Risk Scoring Systems in HLA-Matched Related Allogeneic Hematopoietic Stem Cell Transplantation: A Retrospective Cohort Study

**DOI:** 10.4274/tjh.galenos.2020.2020.0178

**Published:** 2021-06-01

**Authors:** Elifcan Aladağ, Haluk Demiroğlu, Yahya Büyükaşık, Hakan Göker

**Affiliations:** 1 Hacettepe University Faculty of Medicine, Department of Hematology, Ankara, Turkey

**Keywords:** Hematopoietic stem cell transplantation, Risk scoring, Acute leukemia

## Abstract

**Objective:**

Allogeneic hematopoietic stem cell transplantation (AHSCT) is a potentially curative treatment of choice for many hematological diseases. However, there are some transplantation-related risks. Predicting the risk-benefit ratio prior to AHSCT facilitates the choice of conditioning regimens and posttransplant follow-up. Hence, many risk models have been developed. The aim of the present study was to compare 6 different risk models that are clinically used.

**Materials and Methods:**

A total of 259 patients were enrolled in this study. The European Society for Blood and Marrow Transplantation (EBMT), Hematopoietic Cell Transplantation Comorbidity Index (HCT-CI), Age-Adjusted Hematopoietic Cell Transplantation Comorbidity Index (HCT-CI-Age), revised Pretransplant Assessment of Mortality (rPAM), Acute Leukemia-EBMT (AL-EBMT), and Disease Risk Index (DRI) risk models were applied retrospectively.

**Results:**

The AL-EBMT, HCT-CI, and HCT-CI-Age scoring systems were found to be predictive for 2-year overall survival (OS) and 2-year non-relapse mortality (NRM) (2-year OS: AL-EBMT, reference vs. score 8.5-10, HR: 1.3, p=0.035; AL-EBMT, reference vs. score >10, HR: 3.8, p=0.001; HCT-CI: reference vs. score 1-2, HR: 1.4, p=0.018; HCT-CI: reference vs. score ≥3, HR: 2.5, p<0.001; HCT-CI-Age: reference vs. score 1-2, HR: 1.3, p<0.001; HCT-CI-Age: reference vs. score ≥3, HR: 3.2, p<0.001) (2-year NRM: AL-EBMT: reference vs. score 8.5-10, HR: 1.61, p<0.001; AL-EBMT: reference vs. score >10, HR: 3.3, p<0.001; HCT-CI: reference vs. score 1-2, HR: 1.3, p=0.028; HCT-CI: reference vs. score ≥3, HR: 2.3, p=0.011; HCT-CI-Age: reference vs. score 1-2, HR: 1.3, p=0.01; HCT-CI-Age: reference vs. score ≥3, HR: 2.4, p=0.003). In terms of the Kaplan-Meier estimates of 2-year OS and 2-year NRM, the risk scoring system with the highest predictive power was found to be AL-EBMT (2-year AUC: 0.59 and 0.60, respectively). The other scores were not found to be predictive for 2-year OS and NRM.

**Conclusion:**

In the present study at our bone marrow and stem cell transplant center, it has been demonstrated that the HCT-CI, HCT-CI-Age, and AL-EBMT are good predictors of 2-year NRM and OS.

## Abstract (tr)

**Amaç:**

Allojeneik hematopoetik kök hücre nakli (AHKHN) birçok hematolojik hastalıkta kullanılan potansiyel küratif bir tedavi seçeneğidir. Bununla birlikte nakil ilişkili bazı riskler bulunmaktadır. AHKHN öncesi risk-fayda oranının belirlenmesi, kullanılacak hazırlık rejimlerinin seçimi ve nakil sonrası hasta takibini kolaylaştırmaktadır. Bu nedenle birçok risk modeli geliştirilmiştir. Bu çalışmanın amacı, klinik kullanımı olan 6 farklı risk modelini karşılaştırılmasıdır.

**Gereç ve Yöntemler:**

Bu çalışmaya çeşitli hematolojik nedenlerle AHKHN yapılan 259 hasta alınmıştır. Avrupa Kan ve Kemik iliği Nakli Derneği (EBMT), Hematopoetik Hücre Transplantasyon Komorbidite İndeksi (HCT-CI), yaşa göre düzenlenmiş Hematopoetik Hücre Transplantasyon Komorbidite İndeksi (HCT-CI-Yaş), düzenlenmiş nakil öncesi mortalite değerlendirme skoru (rPAM), akut lösemi-EBMT skoru (AL-EBMT) ve hastalık risk indeksi (DRI) risk modelleri geriye dönük olarak uygulanmıştır.

**Bulgular:**

HCT-CI, HCT-CI-Age ve AL-EBMT risk modelleri 2 yıllık genel sağkalım (OS) ve 2 yıllık NRM için prediktif bulundu (2 yıllık OS; AL-EBMT: referans vs skor 8,5-10 HR: 1,3 p=0,035 referans vs skor >10 HR: 3,8 p=0,001, HCT-CI: referans vs score 1-2 HR: 1,4 p=0,018 referans vs skor ≥3 HR: 2,5 p<0.001, HCT-CI-Age referans vs skor 1-2 HR: 1,3 p<0,001 referans vs skor ≥3 HR: 3,2 p<0,001 2 yıllık NRM: AL-EBMT: referans vs skor 8,5-10 HR: 1,61 p<0,001 referans vs skor >10 HR: 3,3 p<0,001 HCT-CI: referans vs skor 1-2 HR: 1,3 p=0,028 referans vs skor ≥3 HR: 2,3 p=0,011 HCT-CI-Age referance vs skor 1-2 HR: 1,3 p=0,01 referans vs skor ≥3 HR: 2,4 p=0,003). İki yıllık OS ve 2 yıllık NRM için, prediktif gücü en yüksek olan test AL-EBMT idi (sırasıyla 2 yıllık AUC; 0,59-0,60). Diğer modeller 2 yıllık OS ve NRM için prediktif değildi.

**Sonuç:**

Referans nakil merkezimizde yapılan bu çalışmada, HCT-CI, HCT-CI-Yaş ve AL-EBMT risk modellerinin 2 yıllık NRM ve OS için iyi birer belirteç olduğu gösterilmiştir.

## Introduction

Allogeneic hematopoietic stem cell transplantation (AHSCT) has been successfully applied as a curative treatment option for many hematological diseases. AHSCT treatment has shown a marked upward trend in the last 20 years [1]. However, this occurred together with an increase in non-relapse mortality (NRM) due to the transplantation. NRM is often related to acute and chronic graft-versus-host disease (GvHD), infections, and transplantation-related toxicities [2]. A decrease in NRM increases the expected overall survival (OS) of patients. Therefore, the selection of a suitable donor and a suitable conditioning regimen to prevent NRM has been a challenge for clinicians [3]. For this purpose, many scoring systems have been developed to evaluate transplantation-related risks. While these predictive risk scores guide clinicians in AHSCT decisions, they also assist in the selection of preparatory regimens and appropriate care after transplantation according to expected risks [4]. These scores can be based on 3 types of systems: a) patient-specific (i.e., CMV serology, donor/recipient age-HLA match, sex match/mismatch, patient comorbidities); b) disease-specific (i.e., underlying disease, disease status, disease stage, time for transplantation); and c) combined patient-, disease-, and center-specific (experience of the transplantation center) [4,5]. A number of retrospective studies have been conducted, especially on 6 different clinical scoring systems [6,7,8,9,10,11]. Of these, the Hematopoietic Stem Cell Transplantation Comorbidity Index (HCT-CI) and its derivative, the HCT-CI-Age Index (HCT-CI-Age), are two scoring systems based on 17 different pretransplant comorbidities of patients. They provide objective and reliable data on the causes of NRM and posttransplant complications based on objective laboratory data and defined morbidities [7,12]. The European Society for Blood and Marrow Transplantation (EBMT) scoring is one of the oldest modeled risk scoring systems and EBMT risk groups have been shown to predict 5-year OS and transplant-related mortality [13,14]. The Acute Leukemia-EBMT (AL-EBMT) scoring system was first developed in 2015 as a machine-learning algorithm to facilitate clinical decision-making in cases of acute leukemias. These calculations provide a 100-day mortality risk for patients. A validation study also demonstrated its strong predictive features for 2-year OS, leukemia-free survival, and 2-year NRM [15]. For the Pretransplant Assessment of Mortality (PAM) score, the age of the patient, donor type, disease risk, preparation regimens, serum creatinine and alanine aminotransferase levels, and forced expiratory volume in one second (FEV1) and diffusing capacity for carbon monoxide (DLCO) values are used. It has been shown to have significant ability to predict especially 2-year OS. However, not many validation studies have been performed [16,17]. A revised PAM (rPAM) scoring system including 5 parameters (age of the patient, donor type, disease risk, FEV1, and patient/recipient CMV serology) was developed in 2015, simplifying the previous one [9]. Another risk assessment model is the Disease Risk Index (DRI). A limited study demonstrated that the DRI risk groups predicted 4-year OS, progression-free survival, incidence of relapse, and NRM, although less effectively so for the last parameter [18,19].

In the present study, we have sought to validate and compare 6 different scoring systems in patients who underwent allogeneic stem cell transplantation in our bone marrow and stem cell transplant center and to demonstrate whether the risk groups predicted 2-year OS and NRM.

## Materials and Methods

All clinical and laboratory data were obtained retrospectively from the electronic medical database system of the Hacettepe University Medical School’s Bone Marrow Transplant Center. The study included 259 patients aged 18 years and older who underwent allogeneic stem cell transplantation between 2006 and 2019 from a matched related donor. Patients whose necessary data for the scoring systems (e.g., patient and donor CMV serology, pretransplant disease assessment) were missing were excluded from the study. Conditioning regimens were categorized as defined by Bacigalupo et al. [20] as either myeloablative (MA) or reduced intensity conditioning (RIC) [21,22].

Prognostic scores were calculated for each patient using the definitions provided in the publications of the respective prognostic indices: the EBMT, AL-EBMT, rPAM score, HCT-CI, HCT-CI-Age, and DRI. The rPAM score was calculated as per online instructions (http://pamscore.org/) and an online calculator was used for the AL-EBMT score (http://bioinfo.lnx.biu.ac.il/~bondi/web1.html) [15]. The DRI was not applicable in patients with aplastic anemia, and the AL-EBMT score was applied only for patients with acute leukemia. The EBMT score of the patients was evaluated in 4 risk groups as 0-2, 3, 4, and >5 [23]. The rPAM score was evaluated in four categories of <17, 17-21, 21-30, and >30 [24]. The HCT-CI-Age score was evaluated in 3 groups as scores of 0, 1-2, and >3 [25]. The 17 comorbidities of the HCT-CI were assessed as previously defined [25,26]. The DRI risk score was analyzed in 4 groups of low, intermediate, high, and very high risk [27]. AL-EBMT scoring was applied according to the expected 100-day mortality value obtained from the online calculator. Patients were divided into 3 risk groups based on 100-day mortality as <8.5%, 8.5%-10%, and >10%.

### Statistical Analysis

The primary endpoint of the study was NRM, accepted as the period between the 0th day of transplantation and mortality due to any causes other than relapse/progressive disease. The secondary endpoint was OS, accepted as the period from the 0th day of transplantation to mortality due to any reason. All analyses were performed using SPSS software (version 20.0; IBM Corp., Armonk, NY, USA).

The 2-year OS ratio of the patients was calculated using Kaplan-Meier curves and the survival difference between the risk groups was compared using the log-rank test. A univariate Cox regression model was used to estimate the impact of the different pretransplant predictive scores on NRM and OS. Cases of p<0.05 were considered statistically significant.

C statistics were used to show the possibility of risk groups to predict the endpoints. A C statistical value of 1 represented the highest concordance, while values of less than 0.5 were considered to signify low concordance.

## Results

### Patient Characteristics

Patient characteristics are shown in Table 1. A total of 259 patients were enrolled. The median age of patients at the time of transplantation was 38 years (range: 18 to 64 years). Performance statuses of all patients were low during transplantation. In 3% of the patients, the Karnofsky performance status was observed to be <80%. A second AHSCT was performed for 10 patients and all the remaining patients were scored according to the values prior to the first AHSCT. While 48.6% of the patients had acute myeloid leukemia, 32.4% had acute lymphocytic leukemia, 7.7% had chronic myeloid leukemia, 6.9% had myelodysplastic syndrome, and 4.2% had non-Hodgkin lymphoma or Hodgkin lymphoma. Transplantations were performed from HLA-matched related donors for all patients. Median follow-up duration after transplantation was 46.6 months. The most common comorbidity was infection (11.9%), followed by diabetes mellitus (9.2%). The least seen side effect was psychiatric disorders (1.5%). Solid tumors and heart valve disease, which have been shown as predictive in HCT-CI scoring, were not seen in any patients.

### Outcomes

### Non-relapse Mortality

Non-relapse mortality was seen in 8.5% of patients. The most common causes of NRM were infection (n=13) and acute GvHD (gastrointestinal system GvHD in 2 patients, GvHD and infection in 6 patients). As shown in Table 2, NRM rates were significantly different among the AL-EBMT, HCT-CI, and HCT-CI-Age risk groups, and those risk scores were found to be positively predictive for 2-year NRM (AL-EBMT: reference vs. score 8.5-10, HR: 1.61, p<0.001; AL-EBMT: reference vs. score >10, HR: 3.3, p<0.001; HCT-CI: reference vs. 1-2, HR: 1.3, p=0.028; HCT-CI: reference vs. score ≥3, HR: 2.3, p=0.011; HCT-CI-Age: reference vs. score 1-2, HR: 1.3, p=0.01; HCT-CI-Age: reference vs. score ≥3, HR: 2.4, p=0.003).

The C statistics of these scoring systems for 2-year NRM were 0.60, 0.51, and 0.52, respectively. The 2-year NRM calculated with the AL-EBMT was 3.1%, 14.3%, and 27% based on low, intermediate, and high risk, respectively. The 2-year NRM values according to the HCT-CI and HCT-CI-Age were 11.2%, 16.2%, and 21.6% and 4.1%, 14.6%, and 21.2% based on low, intermediate, and high risk, respectively (Table 2).

### Overall Survival

**Figure 1:**
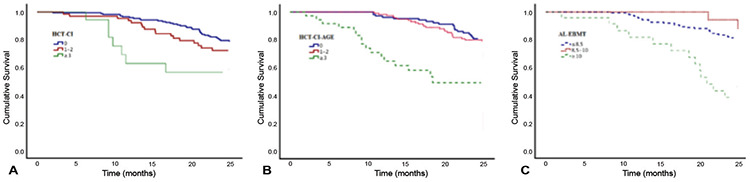
Kaplan-Meier curves of survival in risk groups as defined by HCT-CI **(A)**, HCT-CI-Age **(B)**, and AL-EBMT **(C)**.

The OS values did not show any statistically significant difference when scores were calculated based on the rPAM, DRI, and EBMT scoring systems. In univariate analysis, AL-EBMT, HCT-CI, and HCT-CI-Age risk groups had significant impacts on 2-year OS (AL-EBMT: reference vs. score 8.5-10, HR: 1.3, p=0.035; AL-EBMT: reference vs. score >10, HR: 3.8, p=0.001; HCT-CI: reference vs. score 1-2, HR: 1.4, p=0.018; HCT-CI: reference vs. score ≥3, HR: 2.5, p<0.001; HCT-CI-Age: reference vs. score 1-2, HR: 1.3, p<0.001; HCT-CI-Age: reference vs. score ≥3, HR: 3.2, p<0.001) (Figure 1). C statistics of these scoring systems for 2-year OS were 0.59, 0.52, and 0.56, respectively. While OS was not observed in the group with rPAM scores of 17-24, 2-year OS was found to be significantly higher in patients with rPAM scores of 24-30 and >30 compared to the reference group (rPAM: reference vs. score 24-30, HR 1.8, p=0.037; rPAM: reference vs. score >30, HR 3.6, p=0.012).

## Discussion

The aim of the present study was to test and validate 6 different transplantation risk scores in a patient group. We found high predictive value for 2-year NRM and 2-year OS using the HCT-CI, HCT-CI-Age, and AL-EBMT scores. In our study, the C statistics for both models were rather low (<0.65), and there was a trend toward better predictive capacity for the AL-EBMT compared with the HCT-CI and HCT-CI-Age scores.

The vast majority of risk-based scoring systems are validated with well-defined broad cohorts. Many studies have been published comparing these scoring systems. However, studies generally only evaluate 2 different scores [8,11,28,29]. The present study contributes to the literature in terms of applying 6 different scoring systems for the same patients. To the best of our knowledge, there is only one study in the literature that compared more than 3 risk scoring systems [6]. In that study, 8 different scoring systems were evaluated among 528 patients (EBMT, HCT-CI, Comorbidity-Age, Comorbidity-EBMT, rDRI, PAM, rPAM, and EASIx); among all of them, the models with the highest predictive power for these outcomes were shown to be PAM and rPAM. The PAM score combines patient-, donor-, and disease-related factors. With its update in 2015, replacing DLCO and serum alanine aminotransferase and serum creatinine concentrations with donor/recipient CMV serostatus, the C statistic was similar in the revised and original versions of the PAM scoring system (0.65 versus 0.64) [9]. There are disparate results in the literature for PAM scores. A second study found an association between PAM score and NRM in recipients of MA AHSCT, but not in the subset of reduced intensity recipients, suggesting the lack of utility of the PAM index in patient cohorts with high rates of comorbidities [30,31]. Similarly, we observed that rPAM was predictive for 2-year OS in patients with advanced scores (24-30 and >30). However, the same trend was not observed for 2-year NRM. This may be a result of the tendency toward RIC in our patients.

In this study, the area under the curve (AUC) value of all scoring systems was found to vary between 0.51 and 0.60. These values were found to be low, possibly due to the low number of patients or to the abnormal distribution of patients in the risk groups. Since some components of the scores used in this study were the same for all patients, this might have decreased the predictive power of the scoring systems. For example, donor type, which is used in the EBMT score and may be predictive for NRM, was the same for all of our patients (HLA-matched related donors), and this might have reduced the predictivity of the model for NRM [13]. Likewise, we know that seropositive patients receiving grafts from seropositive donors have improved OS compared to seronegative donors if they have received MA conditioning [32]. The 77% rate of double CMV seropositivity in the patient population may have led to positive selection for the AL-EBMT and rPAM scores. Similarly, the age component was the same in all patients (<65 years) for the rPAM score and matched-related siblings were the donors in all cases, and this caused the majority of patients to be grouped in the low-risk group (score: 17). This might have reduced the predictive power of the rPAM model in this patient group. In addition, this model provides a higher predictive difference in patients undergoing a MA conditioning regimen according to the literature findings. Though subgroup analysis was not performed for patients receiving RIC and MA conditioning regimens, the number of patients transplanted using RIC regimens was observed to be higher.

The novel aspect of this study is that it is a clinical study in which the AL-EBMT model is compared with other scoring systems for the first time. The AL-EBMT score was developed using non-parametric data, unlike all other scoring systems. Although it is predictive for 100-day mortality in patients with acute leukemia, NRM has also been shown to predict leukemia-free survival and 2-year OS. In the validation study performed with 1848 patients in 2017, the hazard ratio of 2-year OS and NRM in intermediate and high risk groups with the reference being the low risk group was 1.3 and 1.24, respectively, and 2.79 and 1.84, respectively (p<0.001; p=0.029) [15]. In the present study, the C statistics of AL-EBMT revealed that the 2-year predictive power was higher both for OS and NRM than that of all other scores. However, this scoring system can only be applied in patients with acute leukemia and this might be considered a source of bias.

## Conclusion

Six different risk scoring systems used for risk assessment prior to AHSCT for patients referred to a tertiary care transplant center were compared in the present study. The AL-EBMT, HCT-CI, and HCT-CI-Age scoring systems were shown to be significantly predictive for 2-year OS and 2-year NRM. These scoring systems are used in many centers since they allow individualized conditioning of the patients for transplantation and guide physicians for better patient follow-up. Future larger multicenter studies are needed to further elucidate the role of these different risk assessment scores and to obtain the most reliable results.

## Figures and Tables

**Table 1:**
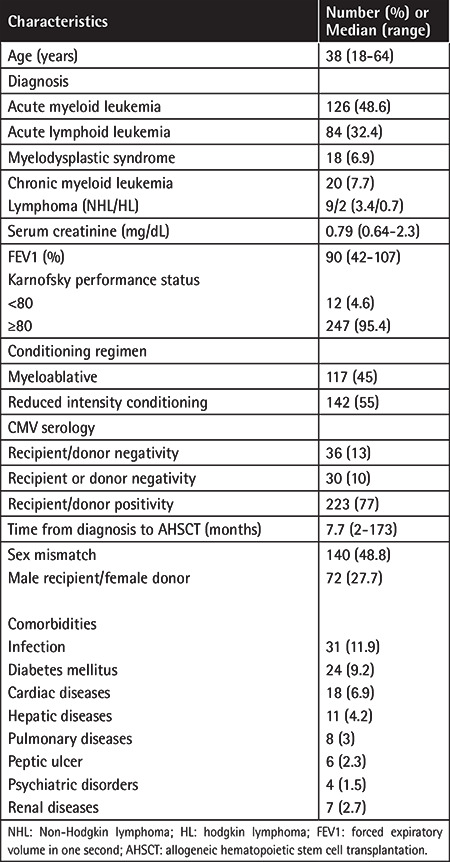
Baseline characteristics of the patient population.

**Table 2:**
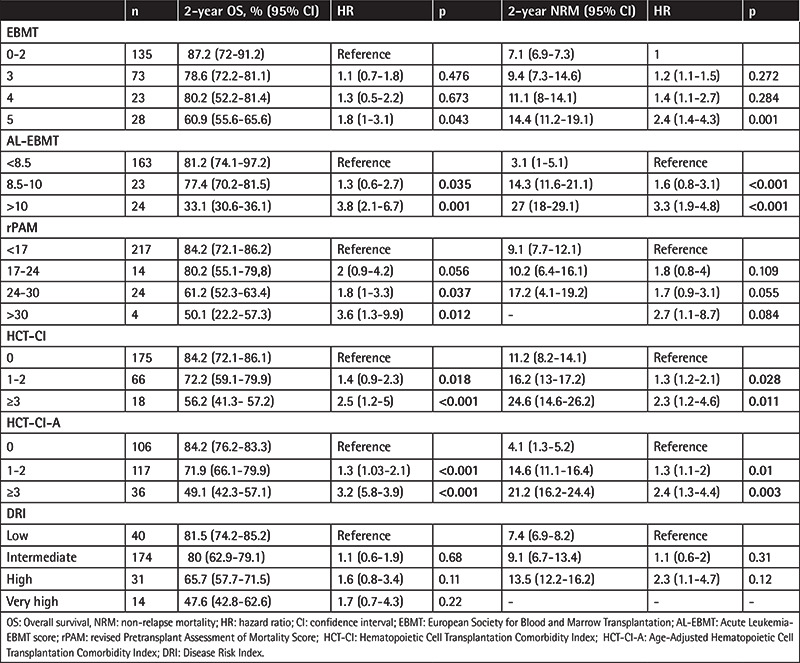
Two-year overall survival and non-relapse mortality according to the risk scoring systems.
